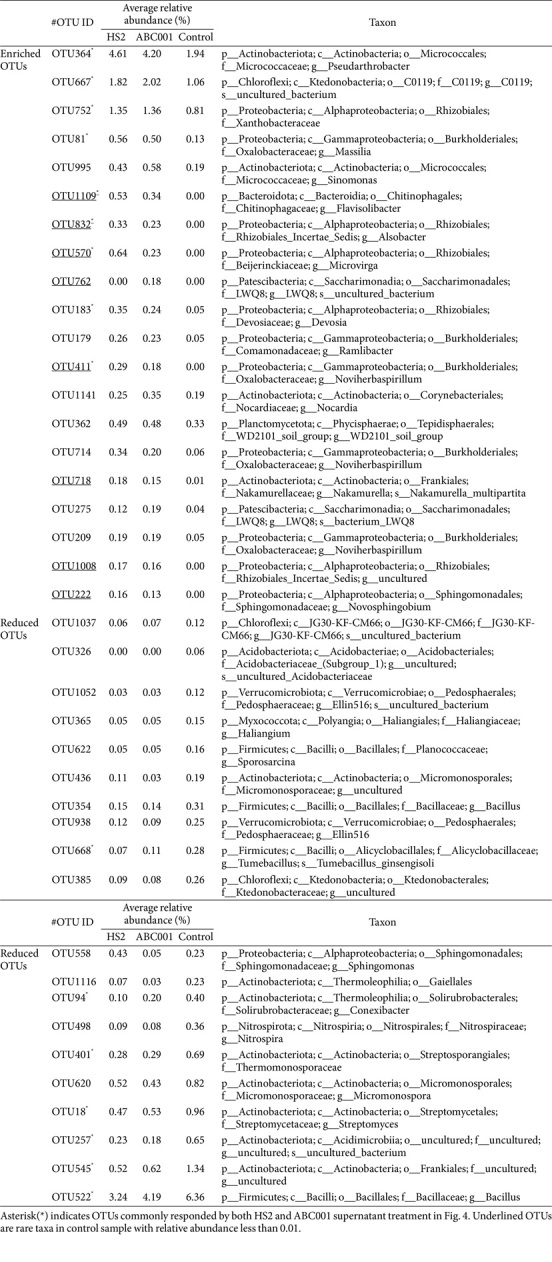# Management of Naturally Occurring Diseases by Supernatant from *Chlorella* Cultures in Pepper

**DOI:** 10.4014/jmb.2502.02004

**Published:** 2025-04-27

**Authors:** Sang-Moo Lee, Hyun Gi Kong, Bongsoo Lee, Yong-Keun Chang, Choong-Min Ryu

**Affiliations:** 1 Molecular Phytobacteriology Laboratory, Infectious Disease Research Center, KRIBB, Daejeon 34141, Republic of Korea; 2 Department of Biosystems and Bioengineering, KRIBB School of Biotechnology, University of Science and Technology, Daejeon 34113, Republic of Korea; 3 Department of Plant Medicine, College of Agriculture, Life and Environment Sciences, Chungbuk National University, Cheongju 28644, Republic of Korea; 4 Department of Microbial and Nano Materials, College of Science and Technology, Mokwon University, Daejeon 35349, Republic of Korea; 5 Department of Chemical and Biomolecular Engineering, Korea Advanced Institute of Science and Technology (KAIST), Daejeon 34141, Republic of Korea

**Keywords:** Biological control, *Chlorella*, microalgae, pepper, microbiome, sustainable agriculture, PGPR

## Abstract

The large-scale culture of *Chlorella*, a genus of microalgae, generates valuable products used to improve human health and produce biofuel. Such commercial applications utilized only the microalgal cells. However, the process produces tons of supernatant waste that require detoxification and disposal. A previous study demonstrated that cell-free supernatants from *Chlorella fusca* culture primes plant immunity in the model plant *Arabidopsis thaliana*, suggesting its potential for use as a bioprotectant. The current study investigated the effects of treatment with *Chlorella* supernatant on crop plants in an agricultural setting. Supernatants from *Chlorella* sp. strains HS2 and ABC001 were drench-applied to pepper (*Capsicum annum* L.) seedlings under greenhouse and field conditions. The disease control capacity and growth of plants were evaluated, as well as the diversity of the rhizosphere microbiota. Application of either supernatant reduced the severity of bacterial leaf spot disease caused by *Xanthomonas axonopodis* pv. *vesicatoria* and enhanced pepper seedling growth in the greenhouse. Under field conditions, cell-free supernatants of strains HS2 and ABC001 not only reduced the severity of natural-occurring viral and bacterial diseases and insect infestation but also increased fruit yield. Additionally, drenching with *Chlorella* supernatants improved species diversity in the rhizosphere microbiota. The application of *Chlorella* supernatant to pepper therefore offered protection against diverse field pathogens and promoted seedling growth and productivity. Our finding provides insight into novel methods of sustainable agriculture utilizing recycled cell-free waste supernatants from the industrial culture of *Chlorella*.

## Introduction

The use of chemical control methods to manage plant diseases is widespread in modern agriculture due to their high efficacy and consistent effectiveness [1]. Long-term use of synthetic agrochemicals may cause severe problems to human health and the environment, however, as well as generating resistant strains of microbial pathogens and insect pests. Biological control methods, which employ various organisms to reduce the activity of plant pathogens, are therefore emerging as an alternative means of crop protection [2]. Biological control agents, such as bacteria, fungi, and yeast, act through various mechanisms, including the production of antimicrobial compounds, resource competition, hyperparasitism, and induced host plant resistance [2-10]. Biological control agents also offer other advantages, including ecofriendly pest management, and therefore provide sustainable methods for cost-effective cultivation of crops, thus safeguarding global food security [2, 11].

Specific plant-associated microbes protect plants against diverse pathogens through a variety of mechanisms, including the activation of induced resistance and the direct antagonism of pathogens [7, 9, 11]. Induced resistance caused by beneficial microbes such as plant growth-promoting rhizobacteria is an especially attractive biological control strategy, as it has long-lasting effects and confers immunity to a wide range of pathogens, including viruses, bacteria, fungi, and insect pests [12-15]. Notably, plants in which induced resistance mechanisms are activated trigger faster and stronger responses upon encountering a pathogen, a phenomenon known as defense priming [16-20]. Since maintaining a primed state is less costly to plants than sustaining a full defense response, which may interfere with growth by imposing an increased energy demand, induced resistance provides advantages for both disease control and stable crop production [17-19, 21, 22].

*Chlorella* is a genus of eukaryotic photosynthetic microorganisms found in various habitats, including fresh and sea water, air, soil, and plant tissues [23-26]. Species of *Chlorella* generate a variety of economically important products, including biofuel, and thus their large scale cultivation has been intensively studied [24, 27, 28]. The commercial culture of *Chlorella* involves cell-based production systems that generate tons of supernatant, whose detoxification and safe disposal present economic and environmental problems [29]. Our previous work proposed the potential of upcycling *Chlorella* supernatants for a model plant such as *Arabidopsis* [26, 30]. To practically apply the recycling of the algal supernatants in agriculture, it is required to apply mass-cultured industrial *Chlorella* supernatant for diverse crop plant under outdoor field conditions.

In this study, to elucidate the effect of agricultural recycling of waste supernatants (up-cycling) from industrial cultivation of *Chlorella*, we applied supernatants from HS2 and ABC001, high lipid-producing strains of *Chlorella* sp., to pepper plants in the greenhouse and field. Application of HS2 and ABC001 supernatants reduced the occurrence of diverse plant diseases and improved plant growth under greenhouse and field conditions. In addition, beneficial effects of both supernatants on the microbial community in the rhizosphere were observed. Overall, our results demonstrated that upcycling of supernatants from large-scale *Chlorella* cultivation provided a novel strategy of biological control.

## Materials and Methods

### Plant Materials

Pepper (*Capsicum annum* L. cv. Bukwang) seeds were sown onto autoclaved soil-free potting medium (Punong Co. Ltd., Republic of Korea) containing zeolite, perlite, colored dust, and lime (pH range 4.5 to 7.5) in 50-hole plastic trays (28 × 54 × 5 cm). After 7 days, pepper seedlings were transplanted into new round pots (diameter 10 cm, height 8.5 cm) containing autoclaved soil-free potting medium (Punon Horticulture Nursery Medium Low; Punong Co. Ltd.). Plants were grown in an environmentally controlled growth room at 25°C under fluorescent lights (light intensity approximately 7,000 lux) and 12 h light/12 h dark cycles.

### *Chlorella* Cultivation and Application of Cell-Free Supernatant to Pepper Seedlings

*Chlorella* sp. ABC001 (also known as *Chlorella* sp. KR1) and HS2 were cultivated under mixotrophic conditions, as described previously [30]. To avoid bacterial contamination, culture media were filtered prior to culture of *Chlorella* using a 0.45 μm syringe filter. To determine the concentration of *Chlorella* cells, cell numbers were counted using a hemocytometer (INCYTO, Republic of Korea). When the culture reached a concentration of 10^7^ cells ml^-1^ (exponential phase), cells were harvested by centrifugation at 4,000 ×*g* for 10 min to separate the supernatant from the pellet.

To examine whether HS2 and ABC001 supernatants could activate plant immunity against *Xanthomonas axonopodis* pv. *vesicatoria* (*Xav*), the root systems of pepper plants were drenched with 10 ml supernatants from either *Chlorella* sp. HS2 or ABC001 culture 5 and 10 days after transplantation to soil. Supernatant-drenched plants were compared with plants treated at the same time points with 0.5 mM benzothiadiazole (BTH), an inducer of disease resistance, or with BG11 broth (Blue-Green medium) as a control treatment.

### Inoculation of Pathogens

The bacterial pathogen *Xav* was cultured at 28°C in Luria-Bertani (LB) broth overnight. To test induced resistance by *Chlorella* supernatant against *Xav* in pepper, the abaxial surfaces of pepper leaves were pressure-infiltrated with the *Xav* culture (OD_600_ = 0.04) using a needleless syringe. The severity of bacterial disease was scored 7 days after pathogen challenge using a 0 to 5 scale (0: no symptoms; 1: slightly yellow color; 2: chlorosis only; 3: partial necrosis and chlorosis; 4: necrosis of the inoculated area and expanded chlorosis; and 5: complete necrosis of the inoculated area).

### Field Trials

Field trials were conducted at Nonsan or Geumsan-gun, Chungcheongnam-do, South Korea. All necessary permits were obtained from the owners of private land prior to commencement of the field trials. To evaluate the biological control ability of *Chlorella* supernatants under typical agricultural conditions, irrigation with water and fertilizer treatments were managed uniformly across all plots, including the negative control, by the farm owner according to the environmental conditions of the experimental site throughout the entire trial period. Before transplanting, the furrows were covered with black polyethylene plastic film to prevent weed growth. Pepper seedlings were planted 30 cm apart. From 1 month of age, seedlings were irrigated with *Chlorella* sp. ABC001 supernatant, *Chlorella* sp. HS2 supernatant, 0.5mM BTH solution, or 1/10 BG11 broth (100 ml per plant) 3 times per month. Each treatment was replicated four or five times in a randomized block design and consisted of 20 plants per treatment. For the assessment of aphid control, each treatment included 40 plants per replication.

### Evaluation of Natural-Occurring Plant Diseases in the Field

To evaluate the biological control activity of *Chlorella* sp. strains ABC001 and HS2 supernatants under field conditions, we measured the incidences in pepper plants of viral infections and bacterial wilt disease and the severity of aphid infestation 2 months after *Chlorella* supernatant treatments at the Nonsan field site (36° 7' 56.69" North, 127° 7' 45.61"East). The incidence of naturally occurring viral infections or bacterial wilt disease on the leaves or stems was assessed by calculating the percentage of pepper numbers showing viral or wilt symptoms, with 20 plants used per treatment, respectively. Aphid infestation severity was calculated by assessing the proportion of aboveground plant parts (from base to apex) that were infested, using a 0 to 5 scale (0: no infestation; 1: < 25% infested; 2: < 50% infested; 3: < 75% infested; 4: 100% infested; 5: plant death).

### Soil Sampling and Analysis of the Rhizosphere Microbiome in Pepper

For microbiome analysis, rhizosphere soil samples were collected at the Nonsan field site (36° 7' 56.69" North, 127° 7' 45.61"East). Soil samples were collected from nine replicate plants per treatment and three different soils were pooled in a single tube to produce three samples per treatment for microbiome analysis. To collect rhizosphere soil, loosely attached soil on the roots was removed, and only the soil tightly attached to the roots was harvested by vigorous shaking. After then, each rhizosphere soil was filtered through a 2 mm mesh to remove large soil particles and plant tissue. Each sample of rhizosphere soil was suspended in sterile distilled water by centrifugation at 200 rpm for 30min. The soil suspension was then centrifuged at 8,000rpm for 10min and the soil pellet containing the microbiome stored at −80°C until microbial analysis.

Microbial genomic DNA was extracted from rhizosphere soil samples using the FastDNA Spin Kit (MP Biomedicals, USA) and quantified using an Epoch Spectrophotometer (Biotek, USA). PCR amplification was performed using primers targeting the V3 and V4 regions of 16S rRNA genes. The first round of amplification used the primers 341F (5'-TCGTCGGCAGCGTCAGATGTGTATAAGAGACAG-CCTACGGGNGGCWGCAG-3') and 805R (5'-GTCTCGTGGGCTCGGAGATGTGTATAAGAGA-CAGGACTACHVGGGTATCTAATC-3') under the following conditions: denaturation at 95°C for 30 s, annealing at 55°C for 30 s, and extension at 72°C for 5 min. Secondary amplification was performed to attach the Illumina NexTera barcodes using the primers i5-F(5'-AATGATACGGCGACCACCGAGATCTACAC-XXXXXXXX-TCGTCGGCAGCGTC-3'; X indicates the barcode region) and i7-R (5'-CAAGCAGAAGACGGCATACGAGAT-XXXXXXXX-GTCTCGTGGGCTCGG-3'; X indicates the barcode region) under the amplification conditions described above but with eight amplification cycles. PCR products were separated by electrophoresis on 1% agarose gels and visualized using a Gel-Doc system (Bio-Rad, USA). PCR products were purified using the CleanPCR Kit (CleanNA, the Netherlands) and equal concentrations of the purified products were pooled. Nontarget short fragments were removed using the CleanPCR Kit. The quality and size of PCR products were assessed using the DNA 7500 chip on a Bioanalyzer 2100 (Agilent, USA). Pooled amplicons were sequenced at ChunLab, Inc. (Republic of Korea) using the Illumina MiSeq platform, according to the manufacturer’s instructions.

### *In silico* Analysis of Microbiome Structure on Pepper Rhizosphere

The quality of the raw sequences was evaluated using FastQC to determine the low-quality cutoffs for forward and reverse readings. The forward and reverse readings were imported into QIIME2 (v 2020.2) [31] for quality control, diversity analysis, and sequence classification. The quality control function in DADA2 (Callahan *et al*., 2016) was used to cut forward and reverse readouts and noise cancellation, and chimera detection and removal. Alpha diversity estimates for community abundance included the Shannon index and operational taxonomic unit and community uniformity estimates included Pielou’s uniformity. Phylogenetic trees were developed in QIIME2 to estimate beta diversity. The pairwise sample estimates (beta diversity) included the weighted unifrac matrix. Classifications of all sample readings were assigned at the species level using the Silva (v. 138.1) Reference Taxonomy Database [32]. Relative proportions were calculated because changes in proportions at the class level are related to soil conditions.

### Assessment of Pepper Fruit Yield in the Field Trial

To determine the fruit yield, the fruit fresh weight and number of fruits per plant were measured at the Nonsan (36° 7' 56.69" North, 127° 7' 45.61" East) and Geumsan field sites (36° 35' 32.27'' North, 127° 30' 34.75'' East). The fruit fresh weight per plant and number of pepper fruits per 20 plants in a row, with four replicate rows, were measured 16 weeks after transplantation to soil. Only red-colored fruits were harvested for market value. Total yield (g/plant) per treatment was estimated and the total fruit weight per plant was calculated. In addition, the number of fruits per plant in each harvest was recorded, and the total harvest was then calculated as the total number of fruits per plant.

### Statistical Analysis

Data were analyzed by Analysis of Variance (ANOVA) using JMP 4.0 software (SAS Institute Inc., USA). Significant treatment effects were determined based on the magnitude of the F-value (*p* < 0.05). When a significant F-value was obtained, mean separation was performed using Fisher’s protected Least Significant Difference (LSD) test at *p* < 0.05. In the case of ordinal disease severity measurements for *Xav* and aphid infection, statistically significant differences were examined using the nonparametric Kruskal-Wallis test, followed by Dunn’s post hoc test for multiple comparisons usinig R program. (http://www.r-project.org/). Statistical analyses for microbiome data were conducted using the R program and QIIME2. Alpha diversity analysis was performed in R using the Shapiro–Wilk normality test, followed by Student’s *t*-test. For beta-diversity analysis PERMANOVA (permutational multivariate analysis of variance) was used with 999 permutations. Differentially abundant taxa were identified by linear discriminant analysis effect size (LEfSE)[33].

## Results

### Cell-Free *Chlorella* Supernatant Activated Induced Resistance in the Greenhouse

**Fig. 1:**
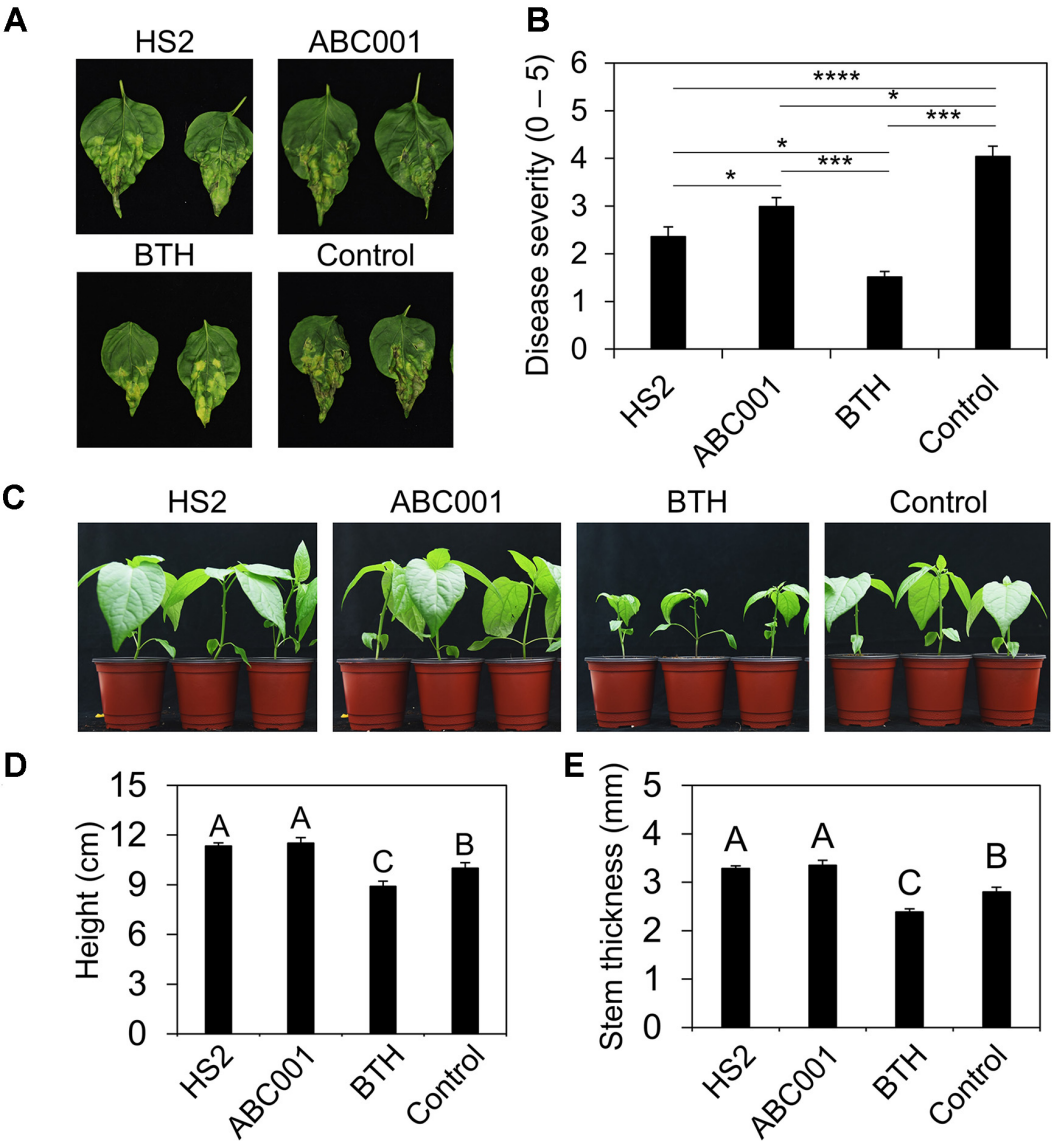
Drenching with *Chlorella* sp. ABC001 and HS2 supernatants enhanced immunity and growth in pepper plants. (A, B) Drenching of pepper plants with *Chlorella* supernatants elicited induced resistance against *Xanthomonas axonopodis* pv. *vesicatoria* (*Xav*). Data shown are means ± standard error of the mean (SEM; *n* = 15 replicates per treatment). Significant difference was noticed by Dunn's test (*p < 0.05; **p < 0.01; ***p < 0.001). (**A**) Photograph showing pepper leaves 1 week after challenge with *Xav*. (**B**) Disease severity measured 1 week after leaf infiltration with *Xav* culture at OD=0.01. (**C**) Foliar application of cell-free supernatants of *Chlorella* sp. HS2 and ABC001 enhanced growth of pepper plants. Photographs were taken 2 weeks after treatment. (**D**) Stem height of pepper plants 2 weeks after treatment with *Chlorella* supernatants. (**E**) Stem width of pepper plants 2 weeks after treatment with *Chlorella* supernatants. HS2: *Chlorella* sp. HS2 supernatant treatment; ABC001: *Chlorella* sp. ABC001 supernatant treatment; BTH: 0.5 mM BTH treatment; Control: BG11 broth treatment. Data shown are means ± standard error of the mean (SEM; *n* = 18 replicates per treatment). Different letters indicate significant differences between treatments according to the Least Significant Difference test (LSD; *p* < 0.05).

To develop a practical method of applying *Chlorella* supernatant on pepper seedlings, we needed to identify specific *Chlorella* strains that offered not only biological control capacity but also advantages for industrial use. Of the many industrial *Chlorella* strains available, we selected *Chlorella* sp. HS2 and ABC001, which exhibit high biological control activity against *Pseudomonas syringae* pv. *tomato* in *Arabidopsis* (*Arabidopsis thaliana*) and *P. syringae* pv. *lachrymans* in cucumber (data not shown). We tested the induced resistance by strains HS2 and ABC001 supernatants in pepper plant against *Xav* in pepper under greenhouse condition (Fig. 1A and 1B). Compared to negative control treatment, drenching with HS2 and ABC001 supernatants reduced the severity of bacterial spot disease by 1.71- and 1.35-fold, respectively (Fig. 1A and 1B). Drenching with BTH, a positive control, reduced the severity of bacterial spot disease by 2.67-fold, compared with the control treatment (Fig. 1A and 1B).

Drenching of two *Chlorella* supernatants also enhanced growth in pepper seedlings (Fig. 1C-1E). Compared with negative control plants, the height of plants drenched with HS2 and ABC001 supernatants increased by 1.13-and 1.15-fold, respectively, and their stem thickness increased by 1.17- and 1.20-fold, respectively (Fig. 1D and 1E). By contrast, drenching with BTH severely restricted the growth of pepper plants (Fig. 1C-1E). Taken together, these results indicated that supernatants from industrial *Chlorella* strains could act as a biological control agent that protected crop plants without imposing any growth penalty.

### Application of *Chlorella* Supernatant Protected Pepper Plants against Naturally Occurring Diseases under Field Conditions

**Fig. 2:**
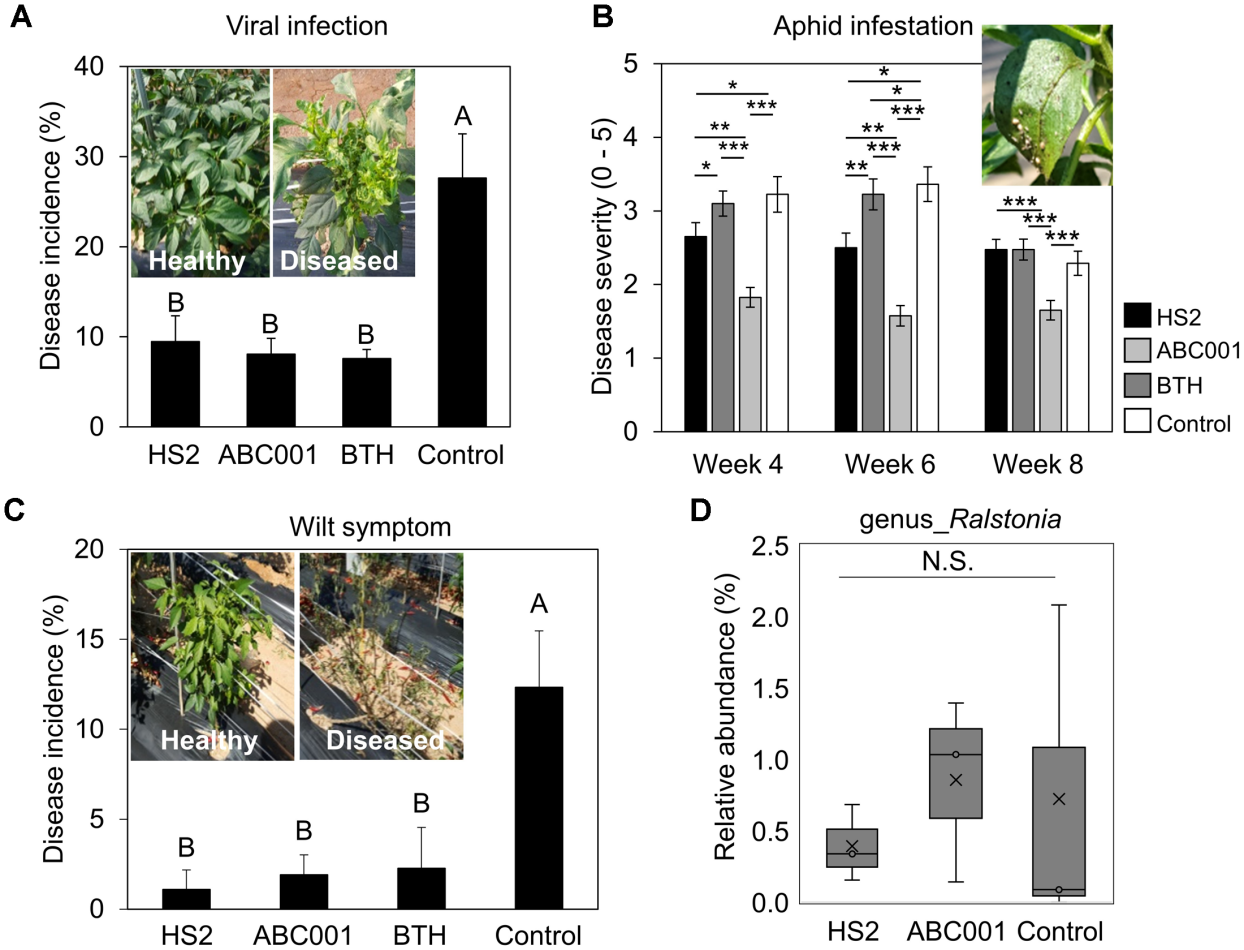
Drenching with ABC001 and HS2 supernatants reduced the occurrence of naturally occurring plant diseases under field conditions. (**A**) Incidence of naturally occurring viral disease in pepper plants treated with *Chlorella* sp. HS2 supernatant, ABC001 supernatant, BTH, or BG11 broth, evaluated 2 months posttreatment. Data shown are means ± standard error of the mean (SEM; *n* = 20 replicates per treatment). The inset photograph shows disease symptoms in pepper plants infected with naturally occurring viruses: heathy plant (left) and diseased plant (right). (**B**) Severity of aphid infestation in pepper plants 4, 6, and 8 weeks post treatment. Data shown are means ± standard error of the mean (SEM; *n* = 40 replicates per treatment). Significant difference was noticed by Dunn's test (*p < 0.05; **p < 0.01; ***p < 0.001). (**C**) Incidence of naturally occurring bacterial wilt symptoms in pepper plants treated with *Chlorella* supernatants. The inset photograph shows the symptoms of naturally occurring wilt in pepper plants: heathy plant (left) and diseased plant (right). Data shown are means ± standard error of the mean (SEM; *n* = 20 replicates per treatment). (**D**) The abundance of *Ralstonia* spp. in pepper rhizosphere soil. N.S.: no significant difference; HS2: *Chlorella* sp. HS2 supernatant treatment; ABC001: *Chlorella* sp. ABC001 supernatant treatment; BTH: 0.5 mM BTH treatment; Control: BG11 broth treatment. Different letters indicate significant differences between treatments according to the Least Significant Difference test (LSD; *p* < 0.05).

To verify the biological control activity of supernatants from industrial *Chlorella* strains under field conditions, the root systems of pepper plants grown at Nonsan, South Korea, were drenched with supernatants from HS2 and ABC001 (Fig. 2A-2D). The drench-application of two supernatants significantly reduced the occurrence of diverse plant diseases caused by virus, bacteria, and insect pest (Fig. 2A-2C). Compared with negative control treatment, drenching with HS2 or ABC001 supernatant reduced the incidence of naturally occurring viral disease by 2.9-fold and 3.4-fold, respectively, whereas treatment with BTH reduced the incidence of viral disease by 3.6-fold (Fig. 2A).

Meanwhile, the infestation of a sucking insect aphid also occurred at 4 weeks after supernatant treatment (Fig. 2B). In negative control treatment, the severity of aphid infestation was scored at 3.2, 3.4 and 2.3 after 4, 6 and 8 weeks, respectively. Similar to BTH, the application of *Chlorella* supernatants, especially HS2 supernatant, reduced aphid infestation in pepper. Aphid infestations in pepper plants treated with HS2 supernatant were rated at 2.7, 2.5, and 2.5 after 4, 6 and 8 weeks, respectively and those in plants treated with BTH were rated at 1.8, 1.6, and 1.7 after 4, 6, and 8 weeks, respectively. Meanwhile, aphid infestations in pepper plants treated with ABC001 supernatant were rated at 3.1, 3.2, and 2.5 after 4, 6 and 8 weeks, respectively.

In addition to the effects on the aboveground parts of plants noted above, treatment with *Chlorella* supernatants reduced the incidence of bacterial wilt disease caused by soil-borne pathogens, mostly *Ralstonia solanacearum* and *R. pseudosolanacearum* (Fig. 2C). Compared with negative control treatment, drenching with HS2 or ABC001 supernatant reduced the incidence of bacterial wilt disease by 11.2-fold and 6.5-fold, respectively. Treatment with BTH reduced the incidence of bacterial wilt by 5.3-fold, compared with the control. The relative abundance of *Ralstonia* spp. was not reduced by either supernatant (Fig. 2D). Taken together, these data showed that cell-free supernatant from high lipid-producing *Chlorella* strains could protect pepper plants against a broad spectrum of pathogens under field conditions. The finding that supernatants from HS2 and ABC001 cultures did not directly inhibit pathogen growth led us to investigate the microbial community in the pepper rhizosphere, in which *Ralstonia* spp. compete with other commensal microbiota.

### Drenching with Cell-Free *Chlorella* Supernatants Modified the Rhizosphere Microbiota

**Fig. 3:**
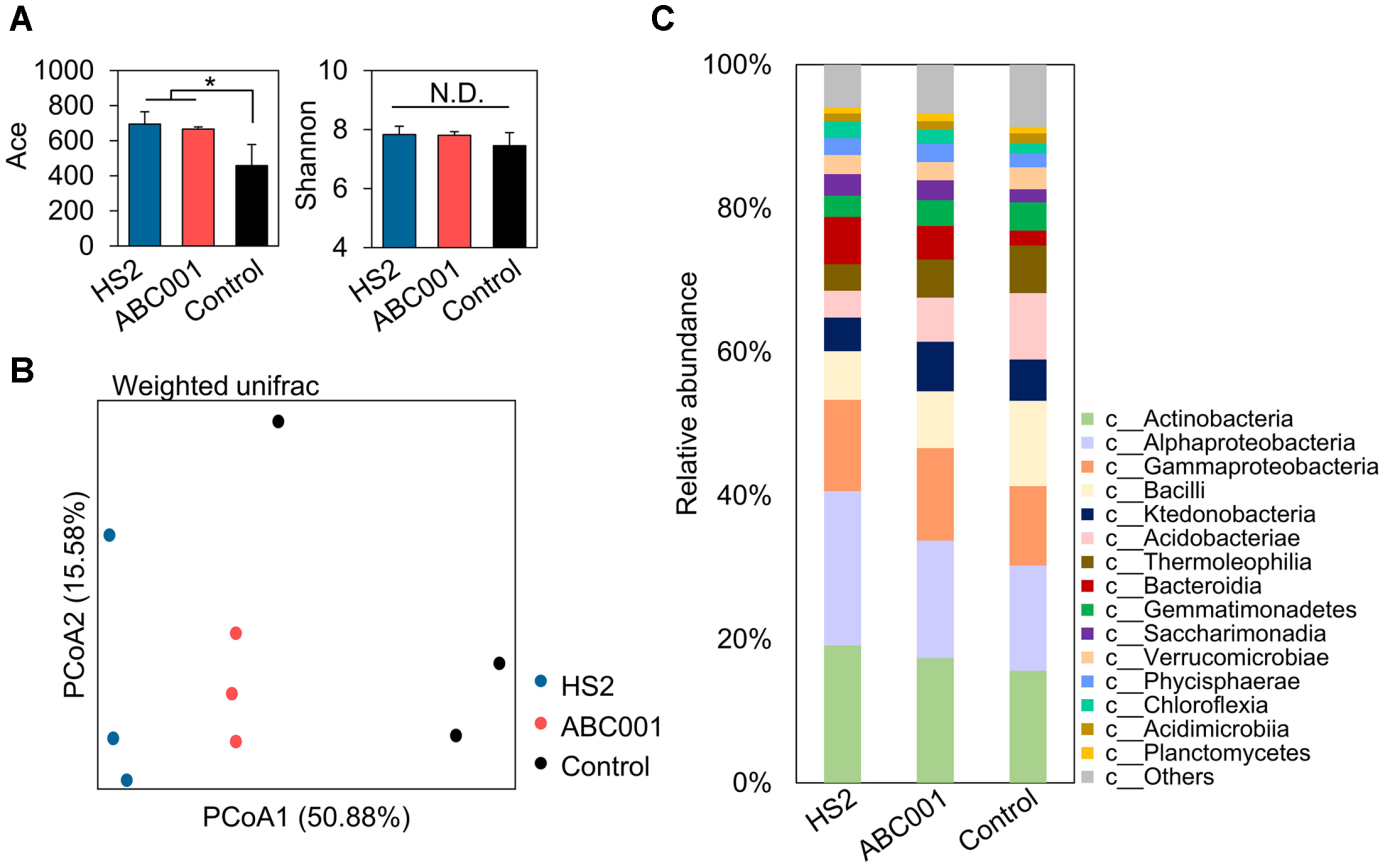
Bacterial community structure in the pepper rhizosphere. (**A**) Changes in the alpha diversity of the pepper rhizosphere microbiome. H: HS2 supernatant treatment; A: ABC001 supernatant treatment; C: BG11 broth treatment. Data shown are means±SEM. Significant difference was noticed by Student’s *t*-test (*p < 0.05; **p < 0.01; ***p < 0.001). (**B**) Twodimensional principal coordinate analysis (PCoA) based on the weighted UniFrac distance metric. Control: BG11 broth treatment; HS2: *Chlorella* sp. HS2 supernatant treatment; ABC001: *Chlorella* sp. ABC001 supernatant treatment. (**C**) Relative abundance of taxa in the pepper rhizosphere at class level. The relative abundance is the mean value, calculated from the abundances in three samples of the pepper rhizosphere soil per treatment.

Previous studies reported that *Chlorella* can modify the structure of the root microbiota in strawberry and *Arabidopsis* [34-36]. We therefore conjectured that strain HS2 and ABC001 supernatant could protect plants against soil-borne pathogens by changing the microbial community associated with pepper roots. We used 16S rRNA gene amplicon sequencing to analyze the rhizosphere microbiome of pepper plants grown under field conditions and treated with the two supernatants (Fig. 3). In total, 249,091 sequences were obtained from all samples. These sequences clustered into 1163 operational taxonomic units (OTUs) with 97% similarity. The rarefaction curves for the samples indicated near saturation of the number of observed OTUs (Fig. S1). Alpha diversity analysis revealed that treatment with the two *Chlorella* supernatants increased the Abundance-based Coverage Estimator (ACE) index, but not the Shannon index, in the rhizosphere bacterial community (Fig. 3A). In addition, principal coordinate analysis (PCoA), based on the weighted unique fraction metric (unifrac) dissimilarity index, showed that the two cell-free supernatant-treated samples clustered separately from the control samples (Fig. 3B). The PCo1 and PCo2 axes accounted for 50.88% and 15.58% of the variance, respectively (Fig. 3B).

**Fig. 4:**
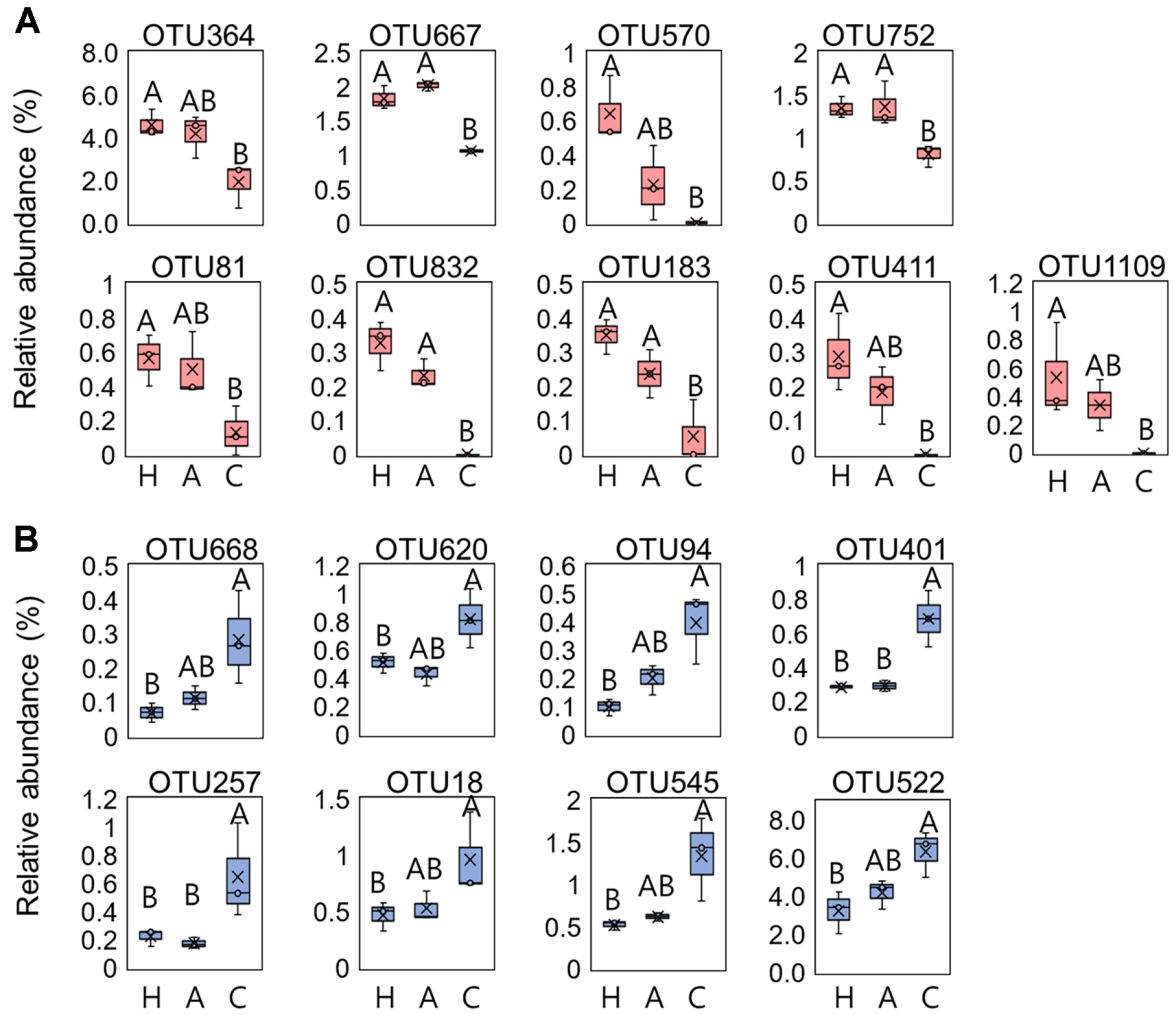
Relative abundance (%) of the operational taxonomic units (OTUs) that identified the keystone taxa common to both the HS2 and ABC001 supernatant treatments. (**A**) Relative abundance of the most discriminating operational taxonomic units (OTUs) enriched in both the HS2 and ABC001 supernatant treatments. (**B**) Relative abundance of the most discriminating OTUs reduced in both the HS2 and ABC001 supernatant treatments. The mean values ± SEs are shown (*n* = 3). Different letters indicate significant differences between treatments according to the Least Significant Difference test (LSD; *p* < 0.05). H: HS2 supernatant treatment; A: ABC001 supernatant treatment; C: BG11 broth treatment. Crosses and middle lines in the boxplot represent the mean and median, respectively, of relative abundance of each OTU.

Relative abundance data indicated that supernatants from HS2 and ABC001 cultures enriched the abundance of the following classes relative to the control treatment: *Actinobacteria*, *Alphaproteobacteria*, *Gammaproteobacteria*, *Bacteroidia*, *Saccharimonadia*, *Phycisphaerae*, and *Chloroflexia* (Fig. 3C). Treatment with the HS2 and ABC001 supernatants enhanced the abundance of gram-negative bacterial groups by 1.08-fold and 1.05-fold, compared with control treatment (Fig. S2). To explore the most discriminating bacterial OTUs in rhizosphere soil treated with *Chlorella* supernatant, we performed a LEfSe analysis (Figs. 4, S3, and S4; Tables 1 and 2). This identified 40 key OTUs that responded to either the HS2 supernatant, or to the ABC001 supernatant, or to both (Tables 1 and 2). In total, nine- OTUs were enriched by treatment with both supernatants, and eight OTUs were reduced (Figs. 4, S3, and S4). Of the OTUs enriched by both supernatants, four (OTU570, 752, 832, and 183) are all from the class *Alphaproteobacteria*, order *Rhizobiales*, while two (OTU81 and 411) belong to the class *Gammaproteobacteria*, order *Burkholderiales* (Fig. 4A, Tables 1 and 2); by contrast, four of the eight OTUs (OTU620, 401, 18, and 545) that were reduced by both supernatants were in class *Actinobacteria* and two (OTU668 and 522) in class Bacilli (Fig. 4B, Tables 1 and 2). In addition, of the 20 OTUs enriched by either HS2 or ABC001 supernatants, eight were classed as rare taxa with a relative abundance of < 0.01% in the control sample; four of these rare taxa (OTU815, 570, 1109, and 832) were enriched following treatment with both supernatants. All the OTUs that were reduced following supernatant treatments were generalists with a relative abundance > 0.01% (Table 1-2).

### Yield increase by Drenching with *Chlorella* Supernatant

**Fig. 5:**
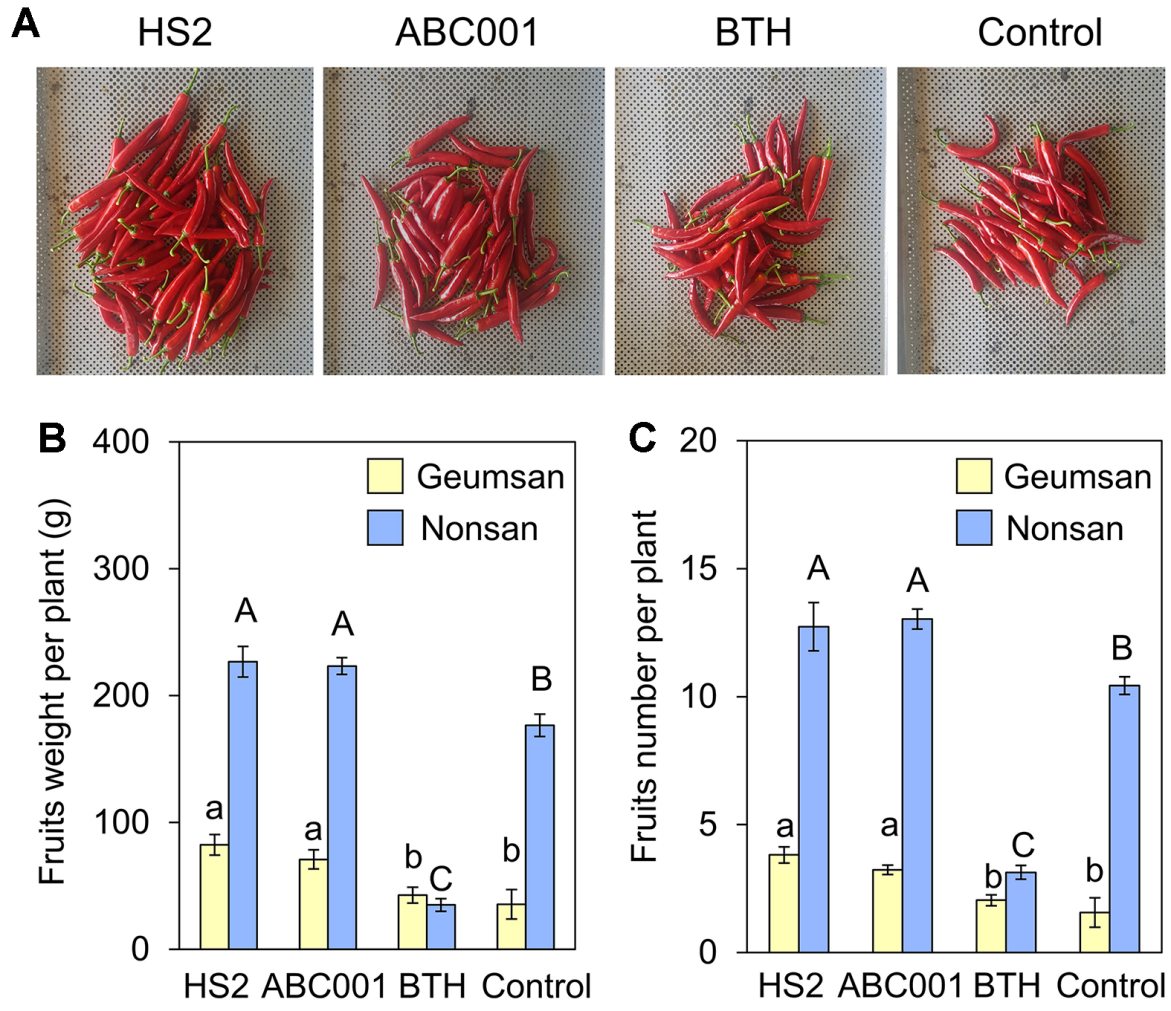
Increase in pepper yield induced by drenching with ABC001 and HS2 supernatants. (**A**) Fruit yield per plant of 20 plants treated with *Chlorella* sp. HS2 supernatant, ABC001 supernatant, BTH, or BG11 broth medium. (**B**) Fruit fresh weight and (**C**) fruit number per plant of 20 plants grown in the field at Nonsan and Geumsan. HS2 sup.: *Chlorella* sp. HS2 supernatant treatment; ABC001 sup.: *Chlorella* sp. ABC001 supernatant treatment; BTH: 0.5 mM BTH treatment; Control: BG11 broth treatment. Different letters indicate significant differences between treatments according to the Least Significant Difference test (LSD; *p* < 0.05).

To investigate the effect on crop yield of treatment with supernatants from the industrial *Chlorella*, we measured fruit weight and number in pepper plants grown in the field at Geumsan, Chungcheongnam-do, Republic of Korea in 2017 and at Nonsan, Chungcheongnam-do, Republic of Korea in 2019, and treated with *Chlorella* supernatants (Fig. 5). Root drenching with HS2 or ABC001 supernatants was associated with a 1.28-fold and 1.26-fold increase, respectively, in the weight of fruit per plant at Nonsan, and with a 2.3-fold and 2.0-fold increase, respectively, at Geumsan by 16 weeks after planting, compared with the control treatment (Fig. 5B). In addition, treatment with HS2 or ABC001 supernatants increased the number of pepper fruits per plant by 2.3-fold and 2.0-fold, respectively, at Nonsan and by 2.4-fold and 2.0-fold, respectively, at Geumsan, compared with the control treatment (Fig. 5C). These results showed that treatment with cell-free supernatant from cultures of two high lipid-producing *Chlorella* strains increased the yield of pepper fruits under field conditions.

## Discussion

*Chlorella* sp. HS2 and ABC001 accumulate high levels of lipids and are therefore cultivated on an industrial scale for biofuel production, generating many tons of supernatant waste that require detoxification [27, 28, 37, 38]. Previously, we proposed that waste supernatant from the industrial culture of *Chlorella* could be upcycled as an agricultural treatment and showed it acted as a bioprotectant and a biostimulant [26, 30]. The use of *Chlorella* supernatants in agriculture therefore presents a practical solution to the economic and environmental issues caused by the disposal of supernatant waste from the industrial-scale cultivation of *Chlorella*. Our current study demonstrated the positive effect of applying supernatant from cultures of HS2 and ABC001 on pepper cultivation under field conditions. In addition, both supernatants enhanced the diversity of microbiota in the rhizosphere, suggesting their potential for use as a prebiotic for crop cultivation [14].

The application of HS2 and ABC001 supernatants protected host plants against a variety of broad-spectrum pathogens, including phytopathogenic bacteria (*Xav* and *Ralstonia* spp.), viruses, and insects (Figs. 1 and 2). Although the mechanisms through which the supernatants enhanced plant protection remain unknown, two scenarios are likely. The first is that the supernatants systemically elicited plant immunity against microbial phytopathogens and insect pests. D-lactic acid, a major component of *Chlorella* supernatants, elicits induced resistance in plants through pathways involving salicylic acid and jasmonic acid and also improves reactive oxygen species (ROS) burst in *Arabidopsis* and tobacco plants [30, 39]. The activation of plant innate immunity by *Chlorella* supernatant cannot be ruled out, given it provided protection against a wide variety of pathogens and insects, and also modified the plant root microbiota. Further evaluation of the effects of supernatants on gene expression related to plant immune responses, as well as on the secretion of root exudates, is required. The second scenario is that modulation of the rhizosphere microbiota by *Chlorella* supernatant protected plants against soil-borne pathogens. Both supernatants enriched the same specific taxa, including *Pseudarthrobacter* (OTU364), *Flavisolibacter* (OTU1109), *Ktedonobacteria* (OTU667), *Microvirga* (OTU570), *Flavisolibacter* (OTU1109), *Massilia* (OTU81), and *Devosia* (OTU183) (Table 1-2). All these taxa have been previously identified as potential biocontrol agents against *Ralstonia solanacearum* [40-47]. Additionally, treatment with either supernatant enhanced the richness of the microbial community in the rhizosphere (Fig. 3A). Such increased diversity in the rhizosphere might thus serve as a competitive barrier that inhibits pathogen invasion near susceptible plant tissues [48-52]. The relative abundance of genus *Ralsonia* did not differ, however, between the control treatment and the HS2 and ABC001 supernatant treatments (Fig. 2D), indicating that the protective effect of the supernatants did not result from direct inhibition of pathogens but was instead elicited indirectly through plant immunity.

Drenching with *Chlorella* supernatants improved the growth of pepper plants under indoor and field conditions (Figs. 1 and 5). *Chlorella* is a photoautotrophic eukaryote that secretes metabolites, including nutrients and organic acids, as well as growth-promoting phytohormones such as auxin, cytokinin and gibberellins [14, 24, 30, 53]. In addition, *Chlorella* engages in symbiotic interactions with various bacteria through exudates containing a variety of beneficial metabolites [54]. The area influenced by these secreted metabolites is known as the microalgal phycosphere. Like the rhizosphere of higher plants, the phycosphere has high microbial species richness [55]. Consistent with earlier studies, supernatants from HS2 and ABC001 cultures increased the abundance of many species of gram-negative bacteria in the root microbiota, including *Stenotrophomonas*, *Beijerinckiaceae*, *Xanthobacteraceae*, *Rhizobium*, *Massilia*, *Flavisolibacter*, and *Sphingomonas* [34-36, 56]. Both supernatants enhanced the abundance of rare taxa, most notably of *Rhizobiales* groups, which are a gram-negative, nitrogen-fixing bacterium, rather than fast-growing generalists (Fig. 4, Tables 1 and 2). In addition, a suspension of *Chlorella* enriches the starch-metabolizing microbiota in strawberry roots [35]. Metabolites in *Chlorella* supernatants may thus not only aid plant growth but also recruit beneficial rare bacteria in the soil of the rhizosphere.

Industrial *Chlorella* cultivation is currently focused on cell harvesting and thus generates many tons of supernatant as a by-product. Disposal of this algal waste can be costly and environmentally harmful. Upcycling of *Chlorella* supernatants in agriculture offers several major benefits. First, its use as a protective treatment to crops would alleviate the concerns related to its economic and environmental costs. The production of 10,000 tons of biodiesel per year from algae cells cultivated in 150-ton bioreactors generates hundreds of tons of supernatant that are currently discarded [29, 30]; if, instead, this supernatant was to be recycled in an agricultural setting, it would be economical and sustainable. Secondly, cell-free *Chlorella* supernatants may prove useful in future agricultural systems since it can be utilized as a safe and easy handling bioactive materials on smart farms. Global warming has led to the emergence of “smart farms”, automated agriculture systems for crop production. In smart farms, pesticides or fertilizers are applied by drip-irrigation, sprinklers, or hydroponic systems [57, 58]. Cell-free *Chlorella* supernatants prevent clogging and can be easily applied in automated systems like sprinklers, drip irrigation, or hydroponics. Thirdly, the application of *Chlorella* supernatants may prevent the emergence of pesticide-resistant pathogens. Abuse and overuse of synthetic pesticides and fertilizers has led to resistant strains of pathogens and disrupted soil microbiota, increasing the risk of disease in agricultural environments [59-61]. *Chlorella* supernatants could be applied as prebiotics to cure this dysbiosis of soil microbial environment.

In conclusion, the application of *Chlorella* supernatants to crop plants may prevent the emergence of pesticide-resistant phytopathogens by enhancing plant immunity and increasing species diversity in soil microbiota. Our results provide new insights into the development of sustainable bioprotectants and biofertilizers by the upcycling of algal waste.

## Supplemental Materials

Supplementary data for this paper are available on-line only at http://jmb.or.kr.



## Figures and Tables

**Table 1:** The list of top 40 key OTUs in rhizosphere microbiome of pepper treated HS2 supernatant.

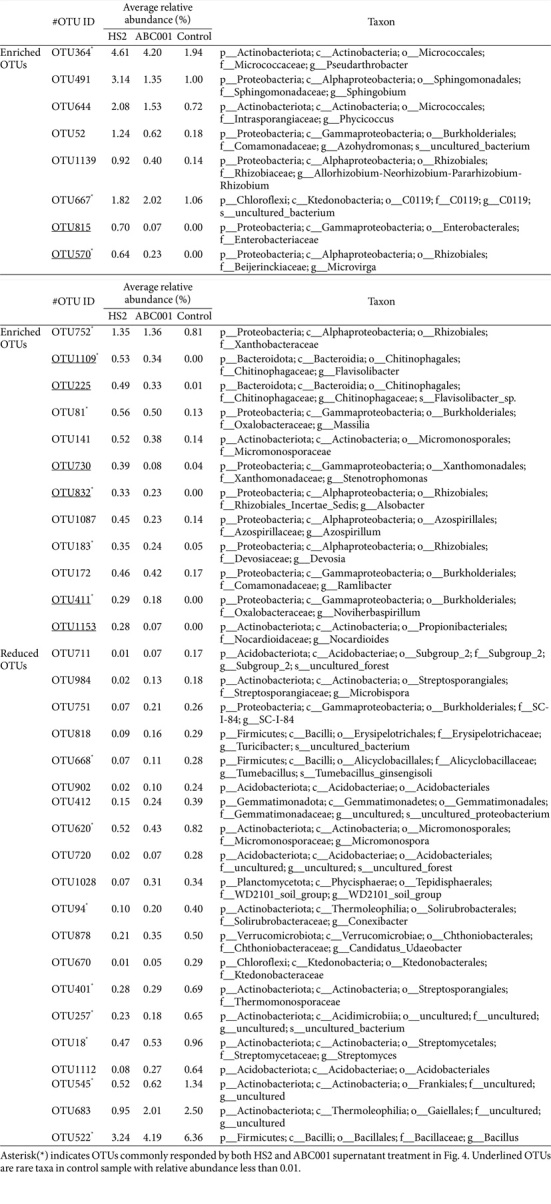

**Table 2:** The list of top 40 key OTUs in rhizosphere microbiome of pepper treated ABC001 supernatant.