# Supplementary material for: Management of Naturally Occurring Diseases by Supernatant from Chlorella Cultures in Pepper
Source: J Microbiol Biotechnol. 2025 Apr 27;35:e2502004. doi: 10.4014/jmb.2502.02004 (PMC12089950; doi:10.4014/jmb.2502.02004)
Supplement: Supplementary file 1 [file jmb-35-e2502004-supple.pdf]

J. Microbiol. Biotechnol. 2025. 35: e2502004

https://doi.org/10.4014/jmb.2502.02004

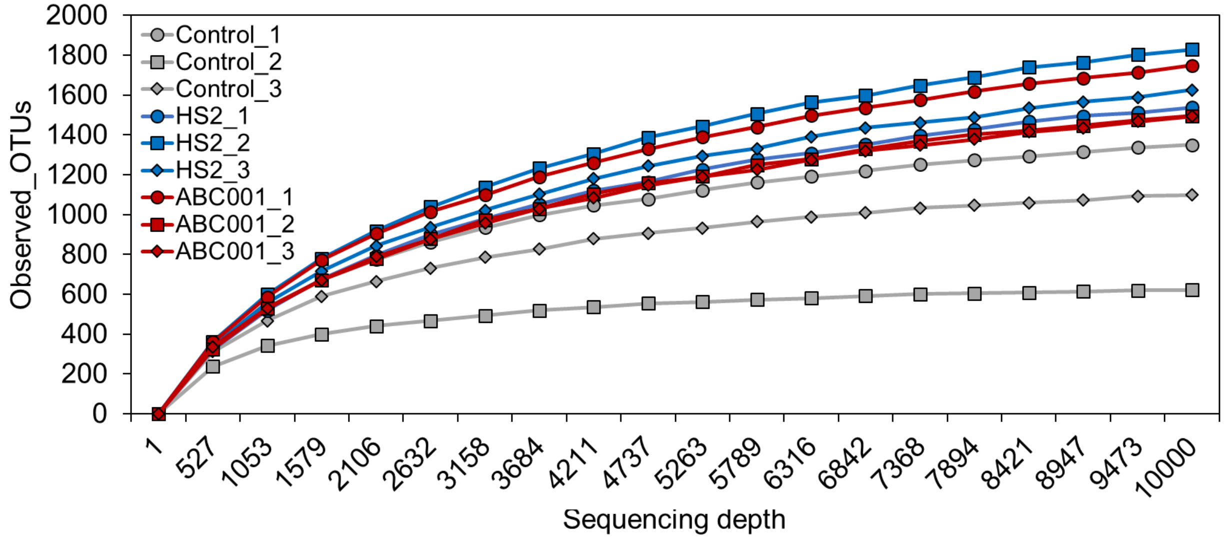

Supplementary Fig. S1. Rarefaction curves for microbiome analysis of pepper rhizosphere soil samples. Control, BG11 medium; HS2, the supernatant of *Chlorella* sp. HS2; ABC001, the supernatant of *Chlorella* sp. ABC001.

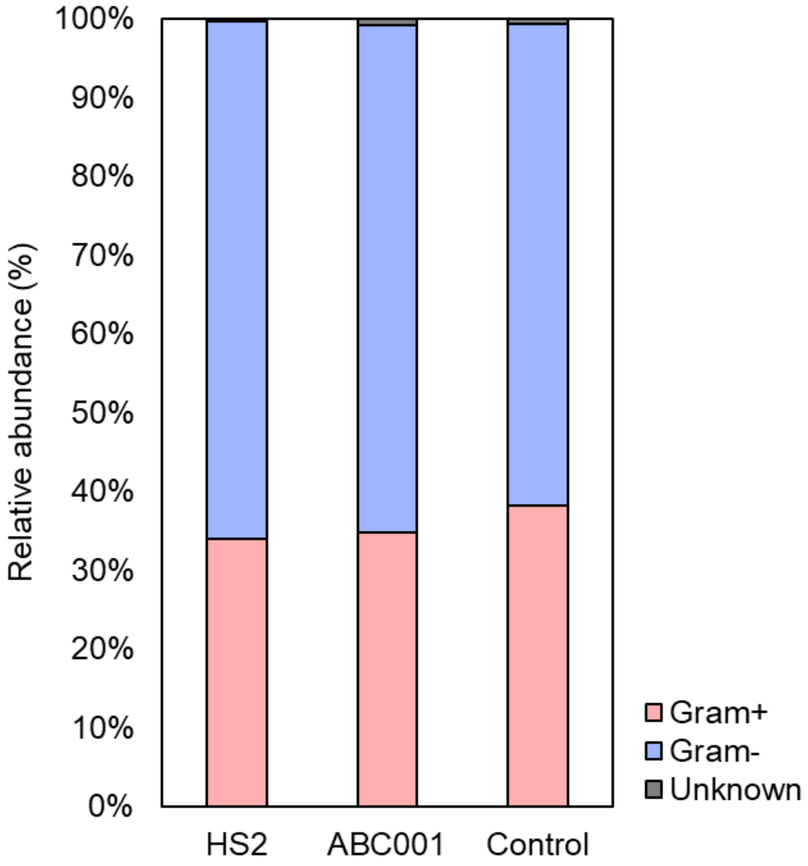

Supplementary Fig. S2. Relative abundance of Gram-positive and Gram-negative bacterial groups in rhizosphere soil of *Chlorella* supernatant treated pepper. Gram+, Gram-positive bacterial groups; Gram−, Gram-negative bacterial groups; Unknown, unclassified groups.

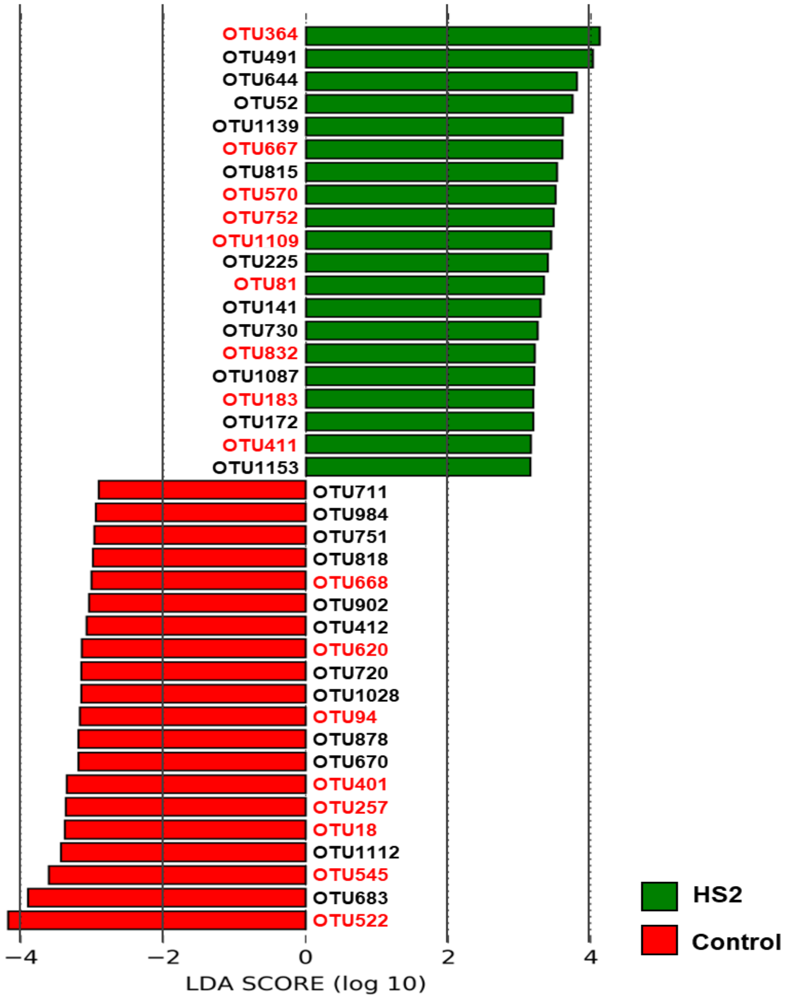

Supplementary Fig. S3. LefSe analysis of the HS2 supernatant-treated pepper rhizosphere microbiota. The most discriminating OTUs showing significant difference in HS2 supernatant treatment (green bar) and control sample (red bar). Red letters indicate the signature OTUs that were commonly enriched or decreased by both HS2 and ABC001 supernatant treatments. Control, BG11 medium; HS2, the supernatant of *Chlorella* sp. HS2

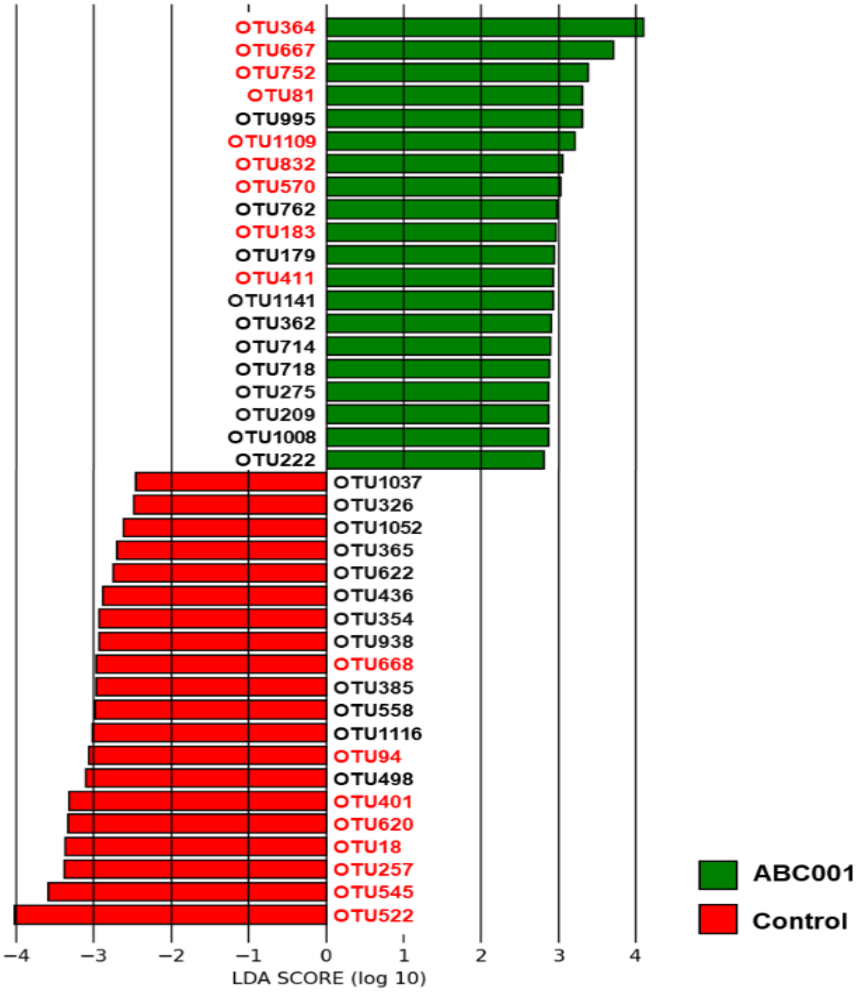

Supplementary Fig. S4. LefSe analysis of the ABC001 supernatant-treated pepper rhizosphere microbiota. The most discriminating OTUs showing significant difference in ABC001 supernatant treatment (green bar) and control sample (red bar). Red letters indicate the signature OTUs that were commonly enriched or decreased by both HS2 and ABC001 supernatant treatments. Control, BG11 medium; ABC001, the supernatant of *Chlorella* sp. ABC001.
